# A new method for real-time monitoring of volatiles in frying fumes using proton transfer reaction mass spectrometry with time-of-flight analyser

**DOI:** 10.1007/s00706-018-2217-8

**Published:** 2018-08-10

**Authors:** Tomasz Majchrzak, Wojciech Wojnowski, Tomasz Dymerski, Jacek Gębicki, Jacek Namieśnik

**Affiliations:** 1 Department of Analytical Chemistry, Faculty of Chemistry, Gdańsk University of Technology, Narutowicza 11/12, 80-233 Gdańsk, Poland; 2 Department of Chemical and Process Engineering, Faculty of Chemistry, Gdańsk University of Technology, Narutowicza 11/12, 80-233 Gdańsk, Poland

**Keywords:** Lipids, Food control, Proton transfer reaction, Mass spectroscopy, Oxidations, Vegetable oil

## Abstract

**Abstract:**

To safeguard the consumers’ well-being, it is necessary to develop novel methods for determination of carcinogens in food, including volatiles generated during frying. The currently used procedures for analysis of volatile fraction of vegetable oils are not based on real-time measurements and thus do not enable the determination of carcinogenic compounds in frying fumes; instead, only the headspace or liquid fraction is sampled. In this article, described is an approach in which proton transfer reaction mass spectrometry with time-of-flight analyser (PTR-TOFMS) was used for real-time monitoring of carcinogenic compounds generated during thermal degradation of rapeseed oil. Using PTR-MS, it was possible to monitor the concentration of known volatile carcinogens according to the International Agency for Research on Cancer (IARC), alongside BTEX compounds, acrolein, and selected aldehydes. Moreover, the applicability of several supervised data analysis methods for the classification of oil samples according to their degree of thermal degradation was presented, with best results obtained using the *k*-nearest neighbours algorithm. Proton transfer reaction mass spectrometry is a powerful technique for the determination of carcinogenic compounds generated during thermal degradation of edible oils. Further investigation of the chemical processes which occur during frying can lead to improvement of food safety.

**Graphical abstract:**

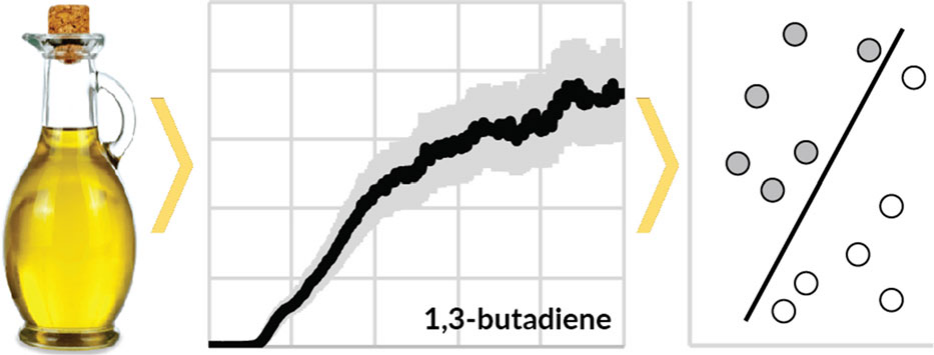

## Introduction

The quality and safety of food products is affected not only by their composition but also by processing. Frying is one of the most commonly used methods of thermal treatment of food. Numerous chemical reactions take place during thermal processing of vegetable oils [1], such as free-radical reaction [2]. These reactions yield volatile organic compounds (VOC), e.g., aldehydes, *trans*-fatty acids, or carboxyl acids [3]. Moreover, thermal degradation of edible oils is associated with cyclization reactions which lead to generation of aromatic compounds, furans, and semi-volatile dimerized fatty acids [4]. Some of these compounds have a detrimental effect on human health and increase the risk of cancer [5]. The International Agency for Research on Cancer (IARC) compiled a list of potentially carcinogenic compounds, which are classified according to the certainty regarding their impact on human health [6]. In particular, Group 1 contains proven carcinogens, some of which are also products of thermal degradation of edible oils.

Numerous methods are currently used to determine the quality of vegetable oil, in particular sensory analysis techniques [7], titration [8], spectrophotometry [9], or gas chromatography [10, 11]. However, only the use of the latter enables the determination of volatile carcinogenic compounds in oil samples, although, due to a relatively long time of a single analysis and sample preparation stage, it is difficult to implement in online monitoring of the heat treatment process. Moreover, methods based on gas chromatography do not allow for real-time monitoring of concentration of volatile compounds in frying fumes. For that reason, there is a need to develop a technique which could be used in online determination of the volatile products of edible oils’ thermal degradation.

In this article, a novel approach for determination of carcinogenic substances in the volatile fraction of vegetable oils is presented. In the proposed solution, proton transfer reaction mass spectrometry (PTR-MS) was used which enables a real-time analysis of headspace composition without the need for extraction and enrichment [12]. The use of PTR-MS makes it possible to obtain immediate quantitative information regarding all the monitored volatiles. To date, this technique was utilized in determination of geographic origin of olive oil [13] and in detection of oxidation products [14]; however, neither of these studies was the time-of-flight analyser used, nor was the analysis carried out continuously in real time. Also included is an example of application of statistical data analysis which was used for the classification of oil samples according to the risk posed to the consumers’ well-being.

## Results and discussion

Concentration of the following compounds from the Group 1 of the IARC list was monitored: formaldehyde, ethylene oxide, acetaldehyde, ethanol, 1,3-butadiene, and benzene. In addition, compounds belonging to the BTEX group: toluene, ethylbenzene, and xylene were also determined as their presence was reported both in edible oils [15] and in rooms where fried food is served [16]. Aldehydes are commonly regarded as indicators of vegetable oils’ quality, since they are formed during oxidation [17, 18]. Hexanal is an indicator of the degree of rapeseed oil’s thermal degradation [19]. For that reason, changes in concentration of pentanal, hexanal, and nonanal were also monitored, together with acrolein which is one of the most toxic compounds produced during frying, as exposure might lead to irritation of respiratory system and necrosis of tissues. There is a need to develop a good analytical procedure for the determination of this compound as it is very volatile, reactive, and prone to polymerisation [20]. It was not possible to distinguish ethylene oxide from acetaldehyde as well as ethylbenzene from xylenes using PTR-MS, since they have the same parent ion molecular mass; however, the toxicity level of these compounds is similar. It should be noted that, since the proton transfer reaction leads to soft ionisation, in most cases, only tentative identification is possible.

**Fig. 1:**
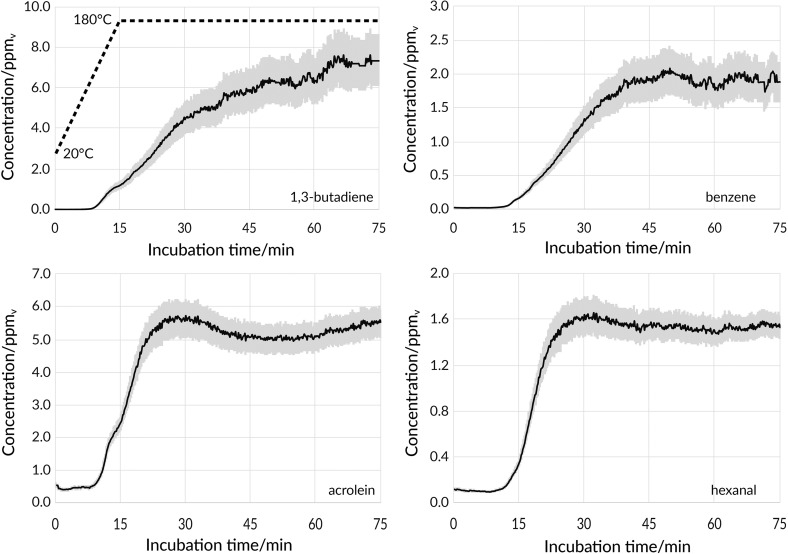
Changes of concentration (ppm_v_, vertical axis) of selected volatiles in the headspace of oil samples over time (minutes, horizontal axis) during heating and frying at 180 °C; SD indicated in grey

Each experiment was comprised of two stages, namely ‘heating’ and ‘frying’. In the former, the increase of concentration of the measured compounds in the volatile fraction was caused by the shift of reaction intensity and evaporation of volatiles from the oil to the gaseous phase. Both reaction intensity and evaporation are dependent on temperature, and so, concentration of the monitored substances in the sample’s headspace increased during this stage. Other parameters affecting the composition of the volatile fraction were the dimensions of the vessel in which the oil was placed—both due to the exchange surface and the volume of gas above the oil—as well as the flow rate of the carrier gas. It should be noted, however, that these parameters were constant during the experiment and similar for each of the analysed samples. In Fig. 1, depicted are the results for selected substances. The concentration of a majority of measured volatiles, including hexanal, acrolein, and benzene, reached a plateau during the second stage which might lead to a conclusion that it is dependent mostly on the temperature of the process, not on its duration. The concentration of other compounds was dependent both on the temperature and duration of the process, and thus increased throughout the experiment. This group contained carcinogenic 1,3-butadiene and BTEX compounds excluding benzene. The concentration of formaldehyde and ethanol peaked 15 min after reaching the frying temperature and then steadily decreased. The discrepancies in the composition of the frying fumes are mostly due to the inability to fully control the conditions of thermal degradation and to slight differences in the composition of oils obtained from different manufacturers.

Listed in Table 1 are the increasing concentrations of monitored substances over time. The concentration of each compound at the beginning of the experiment was lower than 1 ppm_v_, and so, it can be assumed that the sampled rapeseed oil did not contain a significant amount of thermal degradation products, including carcinogenic substances. The highest concentrations at *t* = 75 min was observed for [C_2_H_4_O]H^+^ (ethylene oxide or acetaldehyde), 1,3-butadiene, and acrolein ions: 12.5, 7.3, and 5.5 ppm_v_, respectively. It should be noted that these compounds are the ones with proven detrimental effect on human well-being, including carcinogens, and their elevated concentration in the samples’ headspace is a reason for concern. Moreover, it was confirmed that BTEX compounds are formed during thermal degradation and that aldehydes can be considered indicators of the degree of vegetable oils’ oxidation [18].

**Table 1:** Tentative identification and quantification of monitored substances in volatile fraction of rapeseed oil samples during heating and frying

Entry	Formula	Detected ion formula	Protonated mass/Da	Group
1 Formaldehyde	CH_2_O	[CH_2_O]H^+^	31.018	I Group IARC
2 Ethylene oxide/acetaldehyde^a^	C_2_H_4_O	[C_2_H_4_O]H^+^	45.033	I Group IARC
3 Ethanol	C_2_H_6_O	[C_2_H_6_O]H^+^	47.049	I Group IARC
4 1,3-Butadiene	C_4_H_6_	[C_4_H_6_]H^+^	55.054	I Group IARC
5 2-Propenal	C_3_H_4_O	[C_3_H_4_O] H^+^	57.033	High toxicity to humans
6 Benzene	C_6_H_6_	[C_6_H_6_]H^+^	79.054	I Group IARC, BTEX
7 Pentanal	C_5_H_10_O	[C_5_H_10_O]H^+^	87.080	Quality marker
8 Toluene	C_7_H_8_	[C_7_H_8_]H^+^	93.070	BTEX
9 Hexanal	C_6_H_12_O	[C_6_H_12_O]H^+^	101.096	Quality marker
10 Ethylbenzene/xylenes^a^	C_8_H_10_	[C_8_H_10_]H^+^	107.086	BTEX
11 Nonanal	C_9_H_18_O	[C_9_H_18_O]H^+^	143.143	Quality marker

Entry	Concentration/ppm_v_					
	0 min	15 min	30 min	45 min	60 min	75 min
1 Formaldehyde	0.0286 ± 0.0017^b^	0.801 ± 0.017	1.913 ± 0.042	1.591 ± 0.043	1.455 ± 0.039	1.569 ± 0.039
2 Ethylene oxide/acetaldehyde^a^	0.356 ± 0.030	2.54 ± 0.18	11.90 ± 0.67	11.97 ± 0.89	11.30 ± 0.88	12.5 ± 1.0
3 Ethanol	0.377 ± 42	0.923 ± 0.088	5.81 ± 0.60	4.86 ± 0.46	4.34 ± 0.39	4.52 ± 0.39
4 1,3-Butadiene	0.0181 ± 0.0028	1.18 ± 0.20	4.56 ± 0.63	5.9 ± 1.0	6.3 ± 1.1	7.3 ± 1.3
5 2-Propenal	0.562 ± 0.070	2.46 ± 0.23	5.71 ± 0.53	5.05 ± 0.44	5.06 ± 0.42	5.49 ± 0.44
6 Benzene	0.0234 ± 0.0014	0.164 ± 0.21	1.34 ± 0.20	1.92 ± 0.30	1.77 ± 0.28	1.88 ± 0.30
7 Pentanal	0.0780 ± 0.0089	0.330 ± 0.019	3.09 ± 0.18	3.67 ± 0.19	3.48 ± 0.29	3.76 ± 0.32
8 Toluene	0.0662 ± 0.0078	0.284 ± 0.025	1.92 ± 0.12	2.40 ± 0.21	2.47 ± 0.21	2.77 ± 0.23
9 Hexanal	0.118 ± 0.014	0.350 ± 0.034	1.64 ± 0.16	1.55 ± 0.13	1.50 ± 0.11	1.53 ± 0.10
10 Ethylbenzene/xylenes^a^	0.0147 ± 0.0010	0.0417 ± 0.0026	0.69 ± 0.10	1.06 ± 0.17	1.08 ± 0.17	1.34 ± 0.21
11 Nonanal	0.0654 ± 0.0075	0.1064 ± 0.0060	1.516 ± 0.058	1.72 ± 0.11	1.63 ± 0.12	1.87 ± 0.16

^a^Structural isomers

^b^Standard deviation calculated based on repetitions

**Fig. 2:**
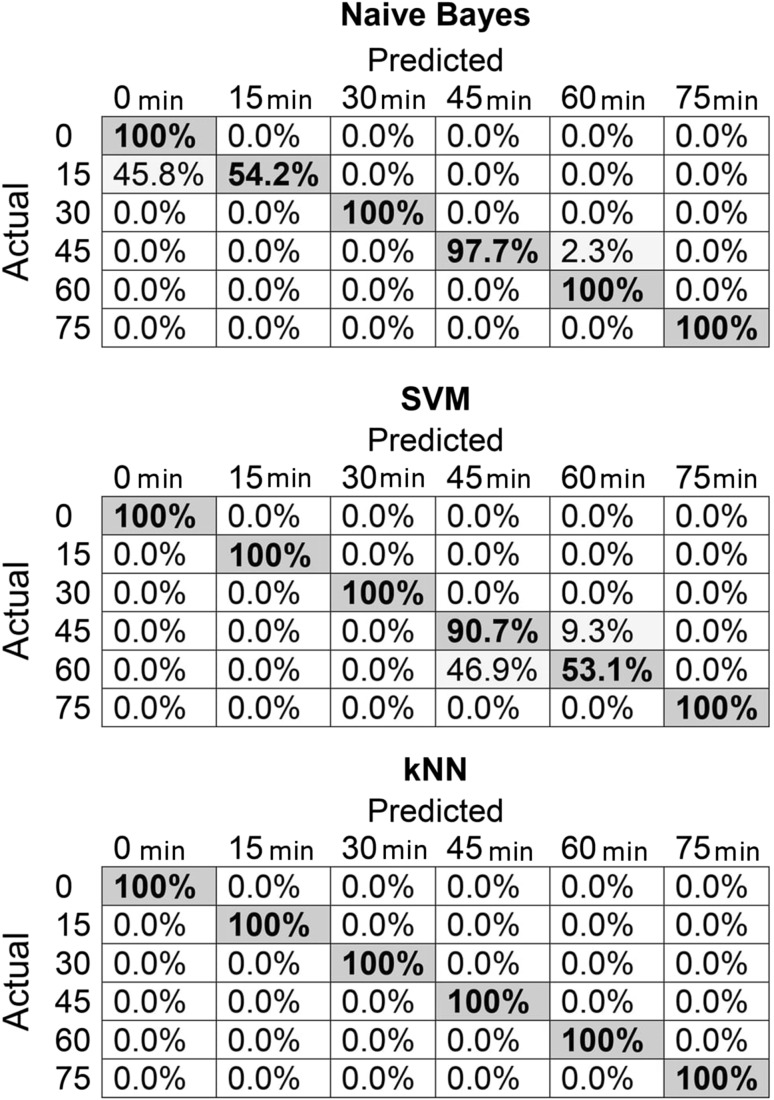
Confusion matrices for selected supervised data analysis methods

In a double-blind test, 70% of data points were used for training the model and 30% for validation. Input data were concentration values at the beginning of the experiment and at consecutive 15 min intervals. Among the selected methods, the lowest classification accuracy was obtained using SVM (90.4%), and the use of *k*-NN allowed for 100% correct classification of input values according to the duration of process (0, 15, 30, 45, 60, and 75 min). In the case of Naïve Bayes method, classification accuracy of 91.8% was obtained. Confusion matrices of the above-mentioned methods are given in Fig. 2.

## Conclusion

Proton transfer reaction mass spectrometry is a powerful technique for determination of carcinogenic compounds generated during thermal degradation of edible oils. Unlike currently used methods, samples can be analysed in real time. Since there is no sample preparation stage, a comprehensive information regarding the composition of the volatile fraction is obtained without propagation of measurement uncertainty. High sensitivity facilitates the detection of compounds at concentration level in the range of tens of ppb_v_. The use of real-time monitoring enables to obtain a more complete information regarding the process of thermal degradation of oil during frying.

As a result of conducted research, selected toxic and carcinogenic substances were identified and determined. The presence of such compounds as acrolein, formaldehyde, 1,3-butadiene, or benzene in the volatile fraction of oil might pose a threat to the consumers’ well-being, as remaining for extended periods of time in rooms where frying takes place and consumption of large amounts of fried food might facilitate the onset of tumours. The presence of BTEX compounds and indicators of vegetable oils’ quality was also confirmed. As a result of frying, the concentrations of monitored compounds in the sample’s headspace exceeded 1 ppm_v_, with the highest observed concentration of 12.5 ppm_v_ in the case of [C_2_H_4_O]H^+^. It was shown that the concentration of some substances was dependent both on temperature and duration of the process, and of some on temperature alone. The factors which determine the presence of volatiles in the oil’s headspace are evaporate from the liquid phase and reactions which take place in high temperatures, mostly oxidation and cyclization.

Also included in this work is preliminary research into the use of supervised data analysis methods aimed at selecting an appropriate statistical method which could in the future be utilized for the classification of oil samples collected during frying in real conditions. It was confirmed that the selected chemical compounds can be used for the development of a statistical model for classification of oil samples based on the degree of thermal degradation. In the future, it is planned to attempt to discriminate between oil samples obtained after an unknown time of frying. Using statistical analysis, it was possible to classify oil samples according to the duration of frying. The use of *k*-nearest neighbours method allowed to obtain the highest classification accuracy.

It is our opinion that further investigation of the chemical processes which occur during frying can lead to improvement of food safety. We are currently developing more application-oriented solutions for the monitoring of carcinogens generated during thermal degradation of vegetable oils.

## Experimental

### Materials

Samples of refined rapeseed oil which is one of the oils most commonly used for frying in Poland were purchased at local distribution centres in Gdańsk, Poland. Five grams of oil were placed in 20 cm^3^ glass headspace vials and sealed with caps lined with silicone-PTFE membrane. To emulate the process of thermal degradation during frying, samples were first heated at a constant rate from 20 to 180 °C which is the frying temperature of rapeseed oil and then incubated at this temperature for an hour in a custom thermostat. In this way, the experiment was divided into two stages: heating of the sample to the frying temperature (15 min) and frying (1 h). The measurement was carried out 15 times.

### Mass spectrometry analysis

To analyse the oil’s headspace in real time, the proton transfer reaction mass spectrometer PTR TOF 1000 Ultra (Ionicon GmbH, Innsbruck, Austria) was used. The distinctive characteristics of this device are its high sensitivity (> 500 cps/ppb_v_) and detection limit below 5 ppt_v_. The use of time-of-flight analyser allows to perform screening without the decrease of detection parameters. During measurement, the transfer line was heated to 70 °C. Mass spectra were recorded every 10 s for a total of 450 spectra per experiment. The details of the device’s operation were described by Jordan et al. [12]. Since the structure and properties of monitored chemical compounds differed significantly, the E/N ratio was set to 130 Td which allowed for a wide range of molecules to be protonated, and only relatively few to become fragmented. For the purpose of quantitative analysis, the following reaction rate constants (*k*; cm^3^/s) were chosen individually for each compound: 2.691 × 10^−9^ (formaldehyde), 2.958 × 10^−9^ (acetaldehyde/ethylene oxide), 2.206 × 10^−9^ (ethanol), 1.817 × 10^−9^ (1,3-butadiene), 3.315 × 10^−9^ (2-propenal), 1.925 × 10^−9^ (benzene), 3.34 × 10^−9^ (pentanal), 2.136 × 10^−9^ (toluene), 3.74 × 10^−9^ (hexanal), 2.319 × 10^−9^ (ethylbenzene/xylenes), and 3.84 × 10^−9^ (nonanal) [21]. Due to the nature of proton transfer reaction, the concentration of ions characteristic for the analytes can be determined without the need for external calibration. Absolute concentration is dependent on the proton transfer rate constant, reaction time, and product and primary ions’ signal [22].

The predominant ions formed as a result of the proton transfer reaction in the proposed conditions are parent ions, although the calculated concentrations not always accurately describe the composition of cooking fumes. For instance, due to the small difference between the proton affinity of water and formaldehyde, and due to the kinetics of the proton transfer reaction, deprotonation of formaldehyde may occur which significantly affects its accurate determination. The presented values pertain to the [CH_2_O]H^+^ ion which may indicate the presence of formaldehyde in the cooking fumes. The actual concentration of formaldehyde is much higher than the reported value.

A dedicated solution was developed to continuously sample the oil’s volatile fraction in a dynamic mode and introduce it into the mass spectrometer.

Carrier gas (ambient air passed through activated carbon filter—Supelpure^®^ HC Hydrocarbon Trap, Sigma-Aldrich) was passed through the headspace of a vial containing the sample at the constant flow rate of 5 cm^3^/min. This is the default gas flow rate resulting from the operating parameters of the vacuum pumps in the PTR-MS device. It is sufficient to maintain aerobic conditions, whilst not as high as to facilitate excessive oxidation. Due to the use of a standard headspace vial and a fixed volume of oil, the headspace volume was comparable between all the oil samples.

A syringe filter with Nylon membrane (0.20 µm) was used to prevent oil droplets from reaching the mass spectrometer’s reaction chamber. IoniTOF v. 2.4.40 software was used to record the spectra and PTR-MS Viewer v. 3.2.3.0 to process the data.

## Data analysis

To classify oil samples according to the degree of thermal degradation and the potential to have a detrimental effect on human health, supervised statistical methods were used, namely Naïve Bayesian model, *k*-nearest neighbours (k-NN), and support vector machines (SVM) with radial basis function (RBF) kernel. The Naïve Bayes model uses Monte Carlo simulations for data classification and was described by Rich [23]. It is not as commonly used as *k*-nearest neighbours, which is among the most popular algorithm for classification of data sets. A comprehensive description of this method can be found in Larose [24]. Theoretical basis of the SVM classifier, in which multi-dimensional data space is divided by a hyperplane, can be found in Boser et al. [25]. Concentration values of selected compounds in the sample’s headspace at the beginning of the experiment (*t* = 0) and after 15, 30, 45, 60, and 75 min were used as input data. Statistical analysis was performed using Orange v.3.3.9 software [26].
